# Performance evaluation of a prescription medication image classification model: an observational cohort

**DOI:** 10.1038/s41746-021-00483-8

**Published:** 2021-07-27

**Authors:** Corey A. Lester, Jiazhao Li, Yuting Ding, Brigid Rowell, Jessie ‘Xi’ Yang, Raed Al Kontar

**Affiliations:** 1 Department of Clinical Pharmacy, College of Pharmacy, University of Michigan, Ann Arbor, MI USA; 2 School of Information, University of Michigan, Ann Arbor, MI USA; 3 Department of Industrial and Operations Engineering, College of Engineering, University of Michigan, Ann Arbor, MI USA

**Keywords:** Decision making, Statistics, Health services

## Abstract

Technology assistance of pharmacist verification tasks through the use of machine intelligence has the potential to detect dangerous and costly pharmacy dispensing errors. National Drug Codes (NDC) are unique numeric identifiers of prescription drug products for the United States Food and Drug Administration. The physical form of the medication, often tablets and capsules, captures the unique features of the NDC product to help ensure patients receive the same medication product inside their prescription bottle as is found on the label from a pharmacy. We report and evaluate using an automated check to predict the shape, color, and NDC for images showing a pile of pills inside a prescription bottle. In a test set containing 65,274 images of 345 NDC classes, overall macro-average precision was 98.5%. Patterns of incorrect NDC predictions based on similar colors, shapes, and imprints of pills were identified and recommendations to improve the model are provided.

## Introduction

Medication errors occur when pharmacy staff count out and give their patient the incorrect medication inside a prescription bottle labeled for a different medication^1–3^. These dispensing errors are potentially harmful to patients, strain the healthcare system, and lead to costly liability fees for pharmacies. In fact, the two most common reasons for legal action against licensed pharmacy staff are the dispensing of the wrong dose and wrong medication with an average paid out claim of nearly $125,000^4^. A multi-site study of dispensing errors in community pharmacies found that incorrect drug, incorrect strength, and incorrect labeling of the prescription vial occurred at a rate 278/109,558 (0.25%) prescriptions^5^. It is critical to provide pharmacy staff with tools to reduce medication errors thereby improving patient safety and reducing healthcare spending. In the pharmacy setting, a fundamental task is to dispense a prescription that ensures the right pill gets to the right patient. Dispensing errors occur when pharmacy staff select a stock bottle of medication, count out, and place the incorrect medication or strength into a prescription vial labeled for a different medication or dose^6–8^. National Drug Codes (NDC) are unique 10-digit, three segment numbers assigned to drugs. However, only the first identifier segment, the manufacturer, is assigned by the United States Food and Drug Administration (FDA). The remaining information on the NDC directory is solely the responsibility of the manufacturer^9^. Relying on humans alone to verify that the physical attributes of medication products for an NDC are insufficient to overcome high workload, interruptions, and limitations of human cognition that are common contributing factors in dispensing errors^6–8^.

Technological advances such as robots that fill medication bottles and barcode scanning to promote getting the right pill into the right vial for the patient are insufficient to eliminate incorrect medication fills and subject to workarounds^10,11^. These technologies are prone to human interaction errors, such as pharmacy staff counting out and labeling the wrong medication after scanning the prescription label and stock medication bottle barcodes. It is difficult to pinpoint the exact number of medication errors as the last comprehensive report was conducted by the Institute of Medicine in 2006^12^. Technological solutions such as barcode scanning do not eliminate errors as a result of human interaction workarounds^11^ and do little to address problems with overburdened pharmacist verifying the prescription. A 2015 study of hospital barcode scanning technology found that barcode scanning did not change the number of errors but rather shifted the types of errors from wrong ingredient to wrong strength and wrong quantity^13^.

In an effort to detect and remedy dispensing errors before they reach the patient, pharmacists perform a verification task by comparing the contents of the filled prescription with an industry reference image. The traditional verification process occurs on site using the physical prescription bottle; however, 24 states currently allow for remote pharmacies in which a pharmacist performs this verification task off-site. Instead, verification by a pharmacist using top-down pictures of the uncapped, filled prescription bottle may occur. Furthermore, a national chain pharmacy recently piloted this remote verification process. Error rates for remote verification are not significantly different from traditional in-person verification; however, the types of errors differ^5^. Remote verification resulted in fewer errors that reached the patient (a near miss) but prescriptions were more likely to contain incorrect directions. In light of the COVID-19 pandemic, further adoption of technology that enables remote verification may occur. Technology assistance of the verification task through the use of machine intelligence (MI) has the potential to detect dangerous and costly pharmacy dispensing errors^3,4,14^.

To assist humans in the verification process, MI models could perform a pill classification task using images taken of medication filled inside a prescription bottle. Previously published studies of pill classification tasks, some spurred by the United States National Library of Medicine’s Pill Image Recognition Challenge, focus on comparing the front and back of individual pills between consumer images with different backgrounds and industry reference images^15–17^. Another study focused on extracting higher-level features (e.g., color, shape, and imprint) of reference pill images using labeled structured data to predict the medication^18^. These previous studies focus on small-scale experimentation of MI models on individual pills. This is achieved by segmenting the background an individual pill then comparing the features to known reference images. In this study, we focus instead on medication dispensing error detection within a pharmacy setting. This requires learning models from a pile of medication inside a prescription vial where image segmentation from the background is impossible as pills are piled on top of each other.

In this paper, we report on training a ResNet-18 deep learning model to predict the labeled features of a medication product using an image showing oral medication inside of a filled prescription vial. The training was done in two stages. First, model fitting occurred by pre-training a ResNet-18 network on the ImageNet dataset^19^. Second, the model was fine-tuned on the dataset showing a pile of pills inside a prescription vial. The objectives are to (1) evaluate model performance of the image classification model that predicts the shape, color, and NDC of prescription medication images, (2) evaluate the reliability of the model predictions, and (3) determine the features of the prescription medication images that lead to incorrect predictions by the model. In doing so, we discuss the implications of utilizing this technology in practice from a trust and safety perspective.

## Results

### Description of the dataset

A dataset of medication images showing a top-down view inside a filled prescription bottle was used to train, test, and validate the classification task. The images were taken after prescription data were transmitted to a robot in a mail-order pharmacy that counted out, weighed, and took a picture of the medication. In practice, these images are used by a pharmacist to verify that the medication inside the prescription bottle is the exact same medication product found on the prescription label for the patient. A total of 65,274 images for 345 NDC oral medication products from 73 manufacturers were included in the test set evaluating the NDC prediction model’s performance. Each NDC medication product has distinct physical characteristics, such as shape, color, and imprints. The majority of NDCs were tablets (80.5%) compared to capsules. The NDC tablets were most often round (55.4%) and oval (21.5%). White pills made up 38.3% of the NDCs, followed by yellow (12.8%), pink (8.4%), and blue (7.0%). NDCs included in the dataset act on the cardiovascular system (40.9%), nervous system (22.3%), and alimentary tract and metabolism (8.7%). The number of images for each NDC ranges from 1 to 1817 with a median of 81.

### Medication image classification

**Fig. 1:**
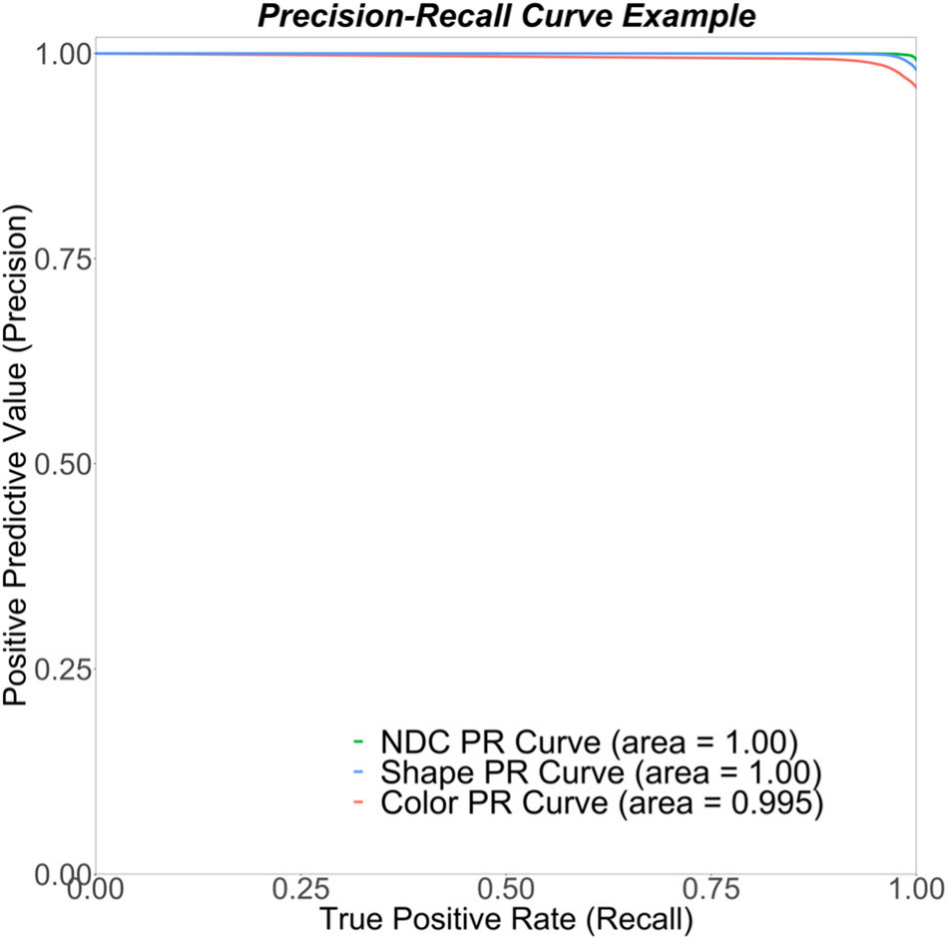
Precision-recall curves for prediction of medication image components. AUC-PR is used as a measure of how well the classifier distinguishes between classes. In our work, the higher the AUC, the better the models are at predicting the NDC, color, or shape. The NDC and the shape model achieved an AUC-PR of almost 1.00 indicating that decision thresholds could be established to minimize both false-positive and false-negative events.

We used the neural network called ResNet-18. The ResNet-18 has experienced success in computer vision for many classification tasks because it solved the gradient vanishing problem and handled the increasing depth of neural networks^20,21^. It consists of an 18-layer residual neural network model that was pre-trained on the ImageNet dataset^19^ and then fine-tuned the parameters using our prescription medication images dataset. First, three ResNet-18 models were trained to predict the NDC, shape, and color, separately. Figure 1 shows a precision-recall (PR) curve for the NDC, shape, and color models. Compared to the traditional receiver operating characteristics (ROC) curve, the PR curve is more appropriate for imbalanced datasets^22^. This curve summarizes the trade-off between the positive predictive value (precision) and the true-positive rate (recall) for various probability thresholds. Overall model accuracies were 99.1%, 94.2%, and 83.6%, respectively. Macro-average precisions of these three models were 0.985, 0.897, and 0.941. As a measure of how well the classifier distinguishes between classes, area under the PR curves (AUC-PR) for each of the three models are reported. In our work, the higher the AUC-PR, the better the models are at predicting the NDC, color, or shape. The NDC model achieved an AUC-PR of almost 1.00 indicating that decision thresholds could be established to minimize both false-positive and false-negative events. Based on the NDC model’s AUC-PR value, as well as the accuracy and the macro-average precision values, compared to the color and shape models, it was selected for further analysis and reporting in this paper.

**Fig. 2:**
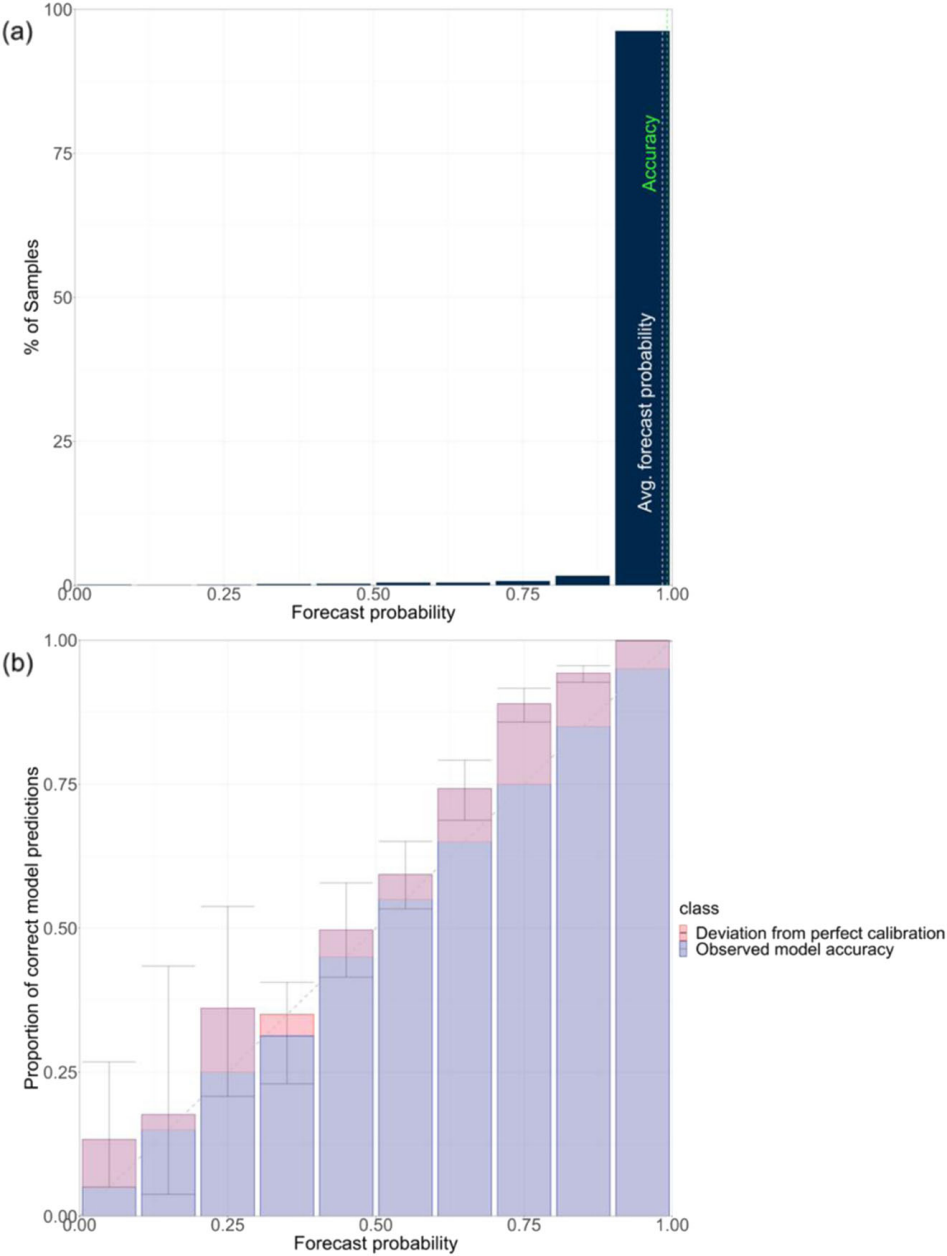
Confidence histogram and reliability diagram for the National Drug Code prediction model on the test dataset. **a** The confidence histogram shows the percentage of samples falling into each forecasted probability bin, while (**b**) the reliability diagram plots the observed predicted probability accuracy against the expected probability, where the range of forecasted probabilities is divided into ten bins (i.e., 0–0.1, 0.1–0.2, 0.2–0.3, etc.). Error bars in the reliability diagram represent 95% confidence intervals for each bin and the numbers of images in each bin are 45, 17, 36, 115, 153, 285, 287, 471, 1063, 62,802, respectively.

The NDC model’s predicted probability distributions for each image were normalized using a softmax operator, such that the sum of all the class probabilities for each image was equal to 1^23^. We used these probability distributions to determine how “confident” it is in the predicted label for each medication image in the test data. We further binned the model’s probabilities into ten probability intervals (i.e., 0.0–0.1, 0.1–0.2, 0.2–0.3, etc.) and plotted these as a confidence histogram and reliability diagram in Fig. 2. A confidence histogram shows the distribution of predicted probabilities across all image observations. In our model, the confidence histogram shows that 96.20% of the model’s predicted probability samples were between 0.9 and 1.0. The model’s overall accuracy and average forecasted probability are also reported to be 99.13% and 98.39%, respectively. A reliability diagram is used to diagnose the degree of calibration of the model with respect to its predicted probability outputs. Calibration error is the difference in the observed prediction accuracy compared to the expected prediction accuracy of the model. Model calibration is important for model interpretation and establishing trustworthiness with end-users^24^.

### Model reliability

The reliability diagram visualizes the model’s observed accuracy in each forecasted probability bin (i.e., the blue bars) and its deviation from perfect calibration (i.e., red bars). The closer the observed accuracy bar in each bin is to the diagonal line, the more reliable the probability estimate is. The dotted line in the reliability diagram represents perfect calibration. For example, in the model predicted probability bin of 0.2–0.3, we would expect the NDC model to be correct in its prediction 25% of the time. The fine-tuned NDC model we used was correct 36.11% of the time when the machine’s predicted probabilities were between 0.2 and 0.3, which is 44.44% higher than expected within this bin. For the NDC model’s predicted probability bin of 0.7–0.8, we would expect the model to be correct in its prediction 75% of the time. The fine-tuned NDC model we used was correct 88.96% of the time at the predicted probability bin of 75; calibration error of 13.96%.

### Comparison of incorrectly predicted medication images

**Fig. 3:**
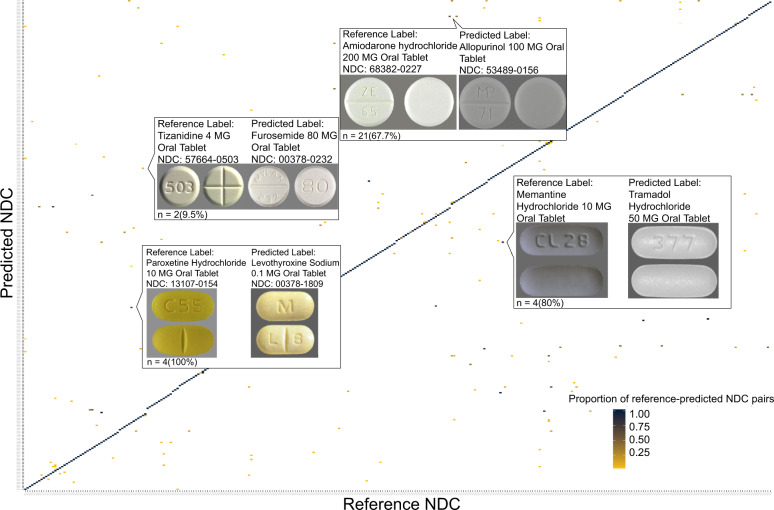
Confusion matrix normalized by proportion of all predicted and reference National Drug Code images. Proportion equal to the predicted National Drug Code (NDC) count for given reference image divided by the count of all reference images of that NDC). 1 = the same NDC was predicted each time; 0 = no cases of reference image being a particular predicted NDC.

In Fig. 3, we report the proportion of NDC predictions made by the machine for each reference NDC in a confusion matrix. The blue diagonal line represents the proportion of correctly predicted NDCs among each reference NDC. Yellow and blue dots outside this diagonal line represent the proportion of incorrect predictions for a given predicted-reference NDC pair. White space indicates that the corresponding pair of reference and predicted NDC never occurred. There were 571 out of 65,274 images (0.9%) in the test dataset that had incorrect NDCs predicted by the NDC model. For example, images showing the medication Amiodarone hydrochloride 200 MG Oral Tablet (NDC = 68382-0227), a medication used to control heart rhythm, were predicted to be Allopurinol 100 MG Oral Tablet (NDC = 53489-0156), a medication used to treat gout, 21 out of 31 times (67.7%) by the model. At this point, we removed 35 images from further analysis as 19 images were not of a top-down view inside a pill bottle and 16 images captured the plastic pill bottles only. Among the remaining 536 images incorrectly predicted, 115 unique NDCs existed and the number of images for each NDC ranged from 1 to 35 with a median of 2.

We next compared the colors and shapes of the predicted NDCs to the corresponding reference NDCs. All of the NDCs labeled as gray and red pills were predicted to be NDCs whose pills are of a different color. The color of predicted NDCs matches the color of pills in the original labels at a 93.8% rate (*n* = 325) for white pill images. We also compared the shape of the predicted NDCs with the labeled shape of the reference NDC. All images of medication in the shape of a triangle (*n* = 186), hexagon (*n* = 289), and trapezoid (*n* = 71) from the test dataset were predicted correctly by the NDC model, while 38.5% (*n* = 169), 30.0% (*n* = 40), 100% (*n* = 1), and 3.4% (*n* = 326) of pills in the shape of a capsule, oval, pentagon (5-sided), and round, respectively, were predicted to be medications of a different shape.

**Fig. 4:**
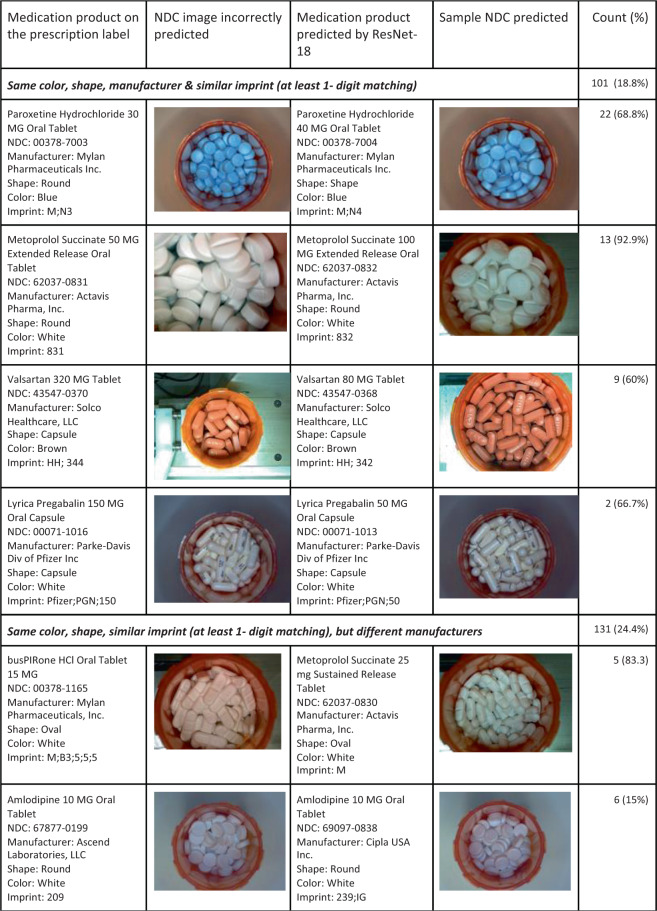
Frequency of the medication National Drug Code incorrectly predicted by the machine sharing similar characteristics. The left-hand columns show an incorrect National Drug Code (NDC) predicted image along with description of the prescription label. The right-hand columns show an example image of the NDC predicted by the machine.

We found that 67.2% (*n* = 360) of predicted NDCs shared the same color and shape with NDCs in the label. Moreover, 18.8% (*n* = 101) medications were predicted to be another medication that shares the same color, shape, manufacturer, and similar imprint (at least 1-character overlap).These medications usually come from the same manufacturer and are of the same ingredients but in different strengths. The average prediction accuracy for these NDCs was 15.8%, which is 87.1% lower than the overall prediction accuracy. Figure 4 shows examples of these incorrectly predicted NDC. The images in the second column show the images that were incorrectly predicted and the right images show an example image of the NDC predicted by the model. Another pattern we identified is when the predicted NDC9 and NDC9 in the label share the same color, shape, similar imprints (at least 1-digit overlap) but are produced by different manufacturers. Out of 536 incorrectly predicted image dataset, 24.4% (*n* = 131) images fall into this category. We also identified similar pairs of color–shape combinations in these images regardless of their imprints. These similar color and shape combinations are white capsule and white oval (*n* = 25, 4.7%), pink capsule and red oval (*n* = 16, 3.0%), brown capsule and gray oval (*n* = 12, 2.2%).

All images we used in the train, validation, and test data come from the same mail-order pharmacy image dataset. However, during the review process, differences in the background of the images were found. For example, the incorrectly predicted image of Valsartan 320 MG Oral Tablet shown in Fig. 4 may be due to the different background of the prescription bottle in the images. We identified 44 out of the 536 (8.2%) images had a relatively different background compared to the rest of images in the incorrectly predicted image set. A randomly selected sample of 536 images from the correctly predicted images set for comparison and only 2 (0.4 %) of them had this distracting background.

## Discussion

To address the lack of MI assistance for the pharmacist verification task, we tested a model to predict the medication product NDC with a macro-average precision of 98.5% and micro-average precision of 99.1% compared to previously published research reporting an unaided pharmacist’s mean accuracy rates of 95.7–99.7%^25,26^. However, more research is needed to determine the accuracy rate of humans verifying images of medication filled rather than viewing the physical contents in-person. Compared to previous work published on identifying prescription medication from images of the front and back views of a single pill, our model achieves greater performance; however, the quantity and quality of images between the different datasets used to train, test, and validate the models are significantly different^15,16,18^. In addition, identifying a medication product by first segmenting out a single pill when an image contains a pile of medication inside a prescription bottle may prove difficult due to the various overlap and postures of the pills.

The NDC model’s AUC-PR of nearly 1.00 compared with a previous experiment in a simulated prescription verification task showed unaided human sensitivity (i.e., detecting an incorrectly filled prescription medication) ranging from 88.2 to 94.2%. Missing an incorrectly filled medication during the verification step is important because of the unnecessary patient harm that can result. For example, a patient expecting an anti-anxiety medication, buspirone, receives a medication for high blood pressure, metoprolol, when the model makes an incorrect decision to approve the medication. Wrong drug and dose errors resulting in professional liability claims against pharmacies result in a nearly $125,000 settlement each time one is filed^4^. Due to the high cost of dispensing errors, technologies should be designed to make reliable decisions that minimize false negative errors in conjunction with a human supervisor^27^. Communicating model uncertainty is a critical factor for improving the trustworthiness of MI advice^28^.

Given that humans have a natural intuition for the meaning of probabilities^24,29^ (e.g., a predicted probability of 75% = a specified outcome occurs 3 out of 4 times), machine probabilities that are discordant with human expectations may impact how a pharmacist interacts with a model’s output to make decisions. In our study, we found that the image classification model tended to be over-calibrated since it was more accurate than expected based on the predicted probabilities produced. When the model is underconfident, it may cause the pharmacist to over-rely on the predicted probabilities since the model is “better than expected.” This can lead the pharmacist to miss an incorrect fill when supervising a system like this because of too much trust in the machine’s predicted probability output.

On the other hand, when the model is overconfident, it may cause the pharmacist to under-rely on the predicted probabilities since the model is “worse than expected.” This can lead a pharmacist to spend more time contemplating the correctness of the medication and increase the cognitive demands of the task because less trust is placed in the machine’s predicted probability output. Eventually, pharmacists may abandon the system altogether because they are skeptical of the machine’s advice. More research is needed to explore how to safely and effectively use MI models to alert pharmacists about potential medication errors, streamline workplace processes, and reduce cognitive demands.

Although the model performs well, a missed incorrectly filled medication that is missed by the model is still problematic. In our review of 571 incorrectly predicted images, we identified pairs of medications with similar color, shape, and imprints. The United States FDA requires imprinting of solid oral dosage forms on drug products for human use^30^. However, medications that share similar color, shape, and imprints with at least one common character accounted for almost 50% of the errors. This problem is especially important when considering the same medication manufacturer for different strengths of the same medication ingredient. For example, Fig. 4 shows that Paroxetine 30MG Tablets are round, blue tablets with the imprint M on one side and N3 on the other. Paroxetine 40MG Tablets made by the same manufacturer are round, blue tablets with the imprint M on one side and N4 on the other. The only other distinguishing characteristic to rely on is the size difference of the two tablets that can be misleading in an image. The error analysis also found that lower quality images (e.g., blurry) and those with different backgrounds were not predicted as well. In these cases, it is reasonable to suspect that humans may have a more difficult time distinguishing between these similar physical features too. It may also be prudent for manufacturers to consider diversifying the physical features of pills in order to help a model, or pharmacist, to distinguish between correctly and incorrectly filled prescriptions. Standardizing the background or segmenting out the vial could be other strategies to improve the model performance.

There are several important limitations to our current analysis. Despite the large number of images and range of NDC products in our dataset, all of the images taken were from a single machine. This decreases the generalizability of the model as performance may degrade with changes in lighting, background, and resolution of the images. The use of individual pill segmentation used in previous research may help^15,16,18^, or, alternatively, a method for segmenting out the top of the prescription vial from the background could be especially helpful^31^. A second limitation is the unbalanced number of images for each NDC. This makes it difficult to determine a cause-and-effect relationship between higher-level pill features, such as color, shape, and imprint and the model’s ability to correctly predict the medication inside the vial. A larger number of samples under more diverse imaging conditions can help to improve the robustness potential utility of the model.

In this paper, we report on the use of a re-trained ResNet-18 model to classify medication NDC products based on a top-down view inside a prescription vial showing a pile of pills with unique colors, shapes, and imprints. Although the model achieves high performance, incorrect predictions by the model can lead to patient harm and increased work for pharmacists. Medications with similar shape, color, and imprints can lead to incorrect model predictions and increase the risk of patient harm. This is especially true for different strengths of the same ingredient produced by the same manufacturer. Manufacturers may consider diversifying the physical features of medication products to minimize the extent of the similarities. Future work focused on the mode for communicating model advice to a human supervisor and measuring the effect on work effort and error detection are critical next steps.

## Methods

### Overview

This is an observational cohort study, in which we re-train a well-known neural network model to perform an image classification task where the contents of the medication inside a prescription bottle prepared by pharmacy staff are predicted. First, we introduce the dataset used and the diversity of medications included. We then describe how we evaluated the model’s performance and how we determined the influence of the medication’s shape and color on the accuracy of the predictions. An exemption from the University of Michigan Institutional Review Board was approved because the study does not meet the definition of research involving human subjects. A combination of Python and R was used for model testing and evaluation.

### Data collection

The medication images used in this study were captured by a commercial medication dispensing robot used at a mail-order pharmacy in the United States. When prescription information is transmitted to the robot for filling, the robot counts out the number of pills into a vial. The prescription label is placed on the side of the vial, an image showing top-down inside the vial is taken, and then it is capped for further order verification by a pharmacist. In addition, the medication contents are weighed and if the weight of the filled prescription is out of pre-specified threshold bounds, then an alert is generated to the pharmacy staff to investigate the medication contents. The process described to obtain these images helps ensure that we have high-quality training, validation, and test data.

Within the dataset of 432,974 images (1024 × 960 resolution), each image is stored with a unique image ID and an 11-digit NDC. The NDC is a numeric code registered with the United States FDA for all prescription and non-prescription medications. We truncated the last two digits of the NDC number as it refers to the quantity of the medication product in the manufacturer’s packaging rather than representing a distinguishing physical appearance of a tablet or capsule. The remaining nine digits represent a unique medication product based on its ingredient, strength, dosage form, and manufacturer. Each NDC product has a distinct physical appearance (e.g., a yellow, round tablet with the imprint, M1).

Data were collected on the physical appearance of the NDC medication for the models using National Institutes of Health PillBox Application Programming Interface^32^, including features of color, shape, size (in mm) of pills, manufacturer, tablet scoring, and imprint. When an NDC reported two colors (e.g., a white and blue capsule) in the labeled data, we assigned a label of “multi-color” for the corresponding NDC to simplify the category labels. We report descriptive statistics of unique NDC in the dataset.

### Model development

We performed three supervised image classification tasks upon this Pharmacy pill Image dataset: 9-digit NDC categories (*n* = 345), pill color categories (*n* = 12), and pill shape categories (*n* = 7) on full-resolution images. We used full-resolution images in our experiments because the contents of the prescription bottle are not segmented from the background of the image. To do this, we used the ResNet-18 deep neural network model proposed by He et al.^20^ for visual recognition-related tasks. The models were implemented using the PyTorch framework^33^. ResNet introduced an additional residual module into traditional deep networks, which helped solve gradient vanishing problems along with handling the increasing number of network layers. The deep structure of ResNet creates a large number of parameters that makes it hard to train a network from the start for our classification task. As a result, we fine-tuned the ResNet-18 network for the medication image dataset, after pre-training on ImageNet^19^. During the modeling prediction process, we used one softmax layer as the output layer to normalize the predicted probabilities and the category corresponding to the maximum probability was selected as the NDC, shape, or color attribute categories for each image. For the three models, the image dataset was separated into training, test, and validation datasets with the same ratio of 7:1.5:1.5 based on the number of labeled images for each outcome category. All three models were fine-tuned for ten epoches with an early stop strategy decided by the result of validation set to prevent overfitting. Other methods including the support vector machines (SVM) and the optical character recognition (OCR) were also considered. However, due to the limitation of the real-world image quality (illumination), image complexity (overlapping pills), and no annotation to help image segmentation, extracting imprints using the traditional OCR method was not adopted. An SVM classifier for NDC was also implemented but was not reported due to low accuracy

### Model evaluation

Three models were developed to predict the 9-digit NDC number, color, and shape of medications inside prescription bottles. The overall accuracy of the model was computed by dividing the number of incorrectly predicted NDC by the total number of images predicted in the test set. We also evaluated the classification results using a PR curve. When the model predicts the NDC number, color, or shape correctly, we consider it as a true-positive prediction. Each point on the PR curve represents the positive predictive value (precision) and the true-positive rate (recall) at a particular classification threshold. As an alternative to the ROC curve, PR curve is more appropriate for datasets with uneven class distributions^22^. The AUC-PR is a widely used measure of the model performance^22,34^. The reported macro-average precision is the average of precision values for each class. This metric helps check the effectiveness of the classifiers on small classes^35^. We conducted additional analyses of the NDC model predictions because it matches the task of the pharmacist most closely. Data analysis of model performance was completed with scikit-learn^36^ and matplotlib^37^ in Python.

Next, a reliability diagram was used to visualize how well the predicted probabilities of the NDC model were calibrated^24^. The diagram shows the percentage correctly predicted NDC by the model against ten equal-size bins of the model’s predicted probabilities (i.e., 0.0–0.1, 0.1–0.2, etc.). The numbers of images in each bin are 45, 17, 36, 115, 153, 285, 287, 471, 1063, 62,802, respectively. Within each bin of predicted probabilities (*x*-axis), the percentage of correct predictions was calculated (*y*-axis). A diagonal reference line represents perfect calibration, where the percentage of correct predictions equals its corresponding predicted probability. The closer the points fall along the diagonal linear line, the better the predicted probabilities by the MI are calibrated. Points above the diagonal line in the plot indicate that the predicted probabilities are too small when they fall into the category, which means the model tends to be correct more often compared to the expected probability. Likewise, when points are below the perfect calibration line, it means the given probabilities are too large and the model tends to be incorrect more often compared to the predicted probability bin. Included in the curve are 95% confidence intervals for each bin. The caret^38^ and tidyverse^39^ packages in R were used for this portion of data analysis.

Finally, we conducted an analysis of the images for all incorrectly predicted NDC, which may have had an impact on the performance of classifiers, and reported image features. To do this, we manually reviewed all incorrectly classified images by the ResNet-18 model. In this step, we removed any inappropriate images (i.e., not an image showing a top-down view inside a prescription bottle). We then identified and categorized key features of NDC products, including color, shape, and imprints, which led to incorrect NDC predictions by comparing those features to the features of the reference NDC. We also examined the composition of the images for correctly and incorrectly predicted images for additional features, such as the number of pills and background setting.

### Reporting summary

Further information on research design is available in the Nature Research Reporting Summary linked to this article.

## Supplementary information

Reporting Summary

## Data Availability

Image data used in this analysis may be accessible with approval from an institutional review board, University of Michigan, and the mail-order pharmacy. Contact the corresponding author.

## Code availability

The experiments and data analysis were carried out using Python with the following openly available libraries: Pytorch, torchvision, numpy, matplotlib, tqdm, pandas, sklearn. The 18-layer residual neural network models (ResNet-18 models) were pre-trained on the 1000-class ImageNet dataset15 and then fine-tuned with Pytorch using the prescription medication images dataset. ImageNet dataset information is available at http://www.image-net.org/. The tuning code may be available upon request and under an agreement with the University of Michigan. Contact the corresponding author.
