# Author Correction: Performance evaluation of a prescription medication image classification model: an observational cohort

**DOI:** 10.1038/s41746-022-00564-2

**Published:** 2022-02-16

**Authors:** Corey A. Lester, Jiazhao Li, Yuting Ding, Brigid Rowell, Jessie ‘Xi’ Yang, Raed Al Kontar

**Affiliations:** 1 Department of Clinical Pharmacy, College of Pharmacy, University of Michigan, Ann Arbor, MI USA; 2 School of Information, University of Michigan, Ann Arbor, MI USA; 3 Department of Industrial and Operations Engineering, College of Engineering, University of Michigan, Ann Arbor, MI USA

**Keywords:** Decision making, Statistics, Health services

Correction to: *npj Digital Medicine* 10.1038/s41746-021-00483-8, published online 27 July 2021

Research reported in this publication was supported by National Library of Medicine of the National Institutes of Health under award number R01LM013624. The contents are solely the responsibility of the authors and do not necessarily represent official views of the National Institutes of Health. The funding was omitted in the original submission and the original article has been corrected.

