# Supplementary material for: Performance evaluation of a prescription medication image classification model: an observational cohort
Source: NPJ Digit Med. 2021 Jul 27;4:118. doi: 10.1038/s41746-021-00483-8 (PMC8316316; doi:10.1038/s41746-021-00483-8)
Supplement: Supplementary file 1 — Reporting Summary [file 41746_2021_483_MOESM1_ESM.pdf]

nature research

Corresponding author(s): Lester, Corey

Last updated by author(s): Feb 10, 2021

Reporting Summary

Nature Research wishes to improve the reproducibility of the work that we publish. This form provides structure for consistency and transparency in reporting. For further information on Nature Research policies, see our Editorial Policies and the Editorial Policy Checklist.

Statistics

For all statistical analyses, confirm that the following items are present in the figure legend, table legend, main text, or Methods section.

n/a	Confirmed	
☐	☒	The exact sample size (*n*) for each experimental group/condition, given as a discrete number and unit of measurement
☒	☐	A statement on whether measurements were taken from distinct samples or whether the same sample was measured repeatedly
☒	☐	The statistical test(s) used AND whether they are one- or two-sided<br>*Only common tests should be described solely by name; describe more complex techniques in the Methods section.*
☒	☐	A description of all covariates tested
☐	☒	A description of any assumptions or corrections, such as tests of normality and adjustment for multiple comparisons
☐	☒	A full description of the statistical parameters including central tendency (e.g. means) or other basic estimates (e.g. regression coefficient) AND variation (e.g. standard deviation) or associated estimates of uncertainty (e.g. confidence intervals)
☒	☐	For null hypothesis testing, the test statistic (e.g. *F*, *t*, *r*) with confidence intervals, effect sizes, degrees of freedom and *P* value noted<br>*Give P values as exact values whenever suitable.*
☒	☐	For Bayesian analysis, information on the choice of priors and Markov chain Monte Carlo settings
☒	☐	For hierarchical and complex designs, identification of the appropriate level for tests and full reporting of outcomes
☒	☐	Estimates of effect sizes (e.g. Cohen's *d*, Pearson's *r*), indicating how they were calculated

Our web collection on statistics for biologists contains articles on many of the points above.

Software and code

Policy information about availability of computer code

Data collection	An image dataset from a mail-order pharmacy was used in the analysis. Each image shows a top-down view inside a prescription bottle filled with pills. Additionally, the US National Library of Medicine Pillbox was used to obtain the physical attribute labels for each NDC attached to the image.
Data analysis	The experiments and data analysis were carried out using Python with the following openly available libraries: Pytorch, torchvision, numpy, matplotlib, tqdm, pandas, sklearn. The 18-layer residual neural network models (ResNet-18 models) were pre-trained on the 1000-class ImageNet dataset15 and then fine-tuned with Pytorch using the prescription medication images dataset. ImageNet dataset information is available at http://www.image-net.org/. The tuning code may be available upon request and under an agreement with the University of Michigan.

For manuscripts utilizing custom algorithms or software that are central to the research but not yet described in published literature, software must be made available to editors and reviewers. We strongly encourage code deposition in a community repository (e.g. GitHub). See the Nature Research guidelines for submitting code & software for further information.

Data

Policy information about availability of data

All manuscripts must include a data availability statement. This statement should provide the following information, where applicable:

- Accession codes, unique identifiers, or web links for publicly available datasets
- A list of figures that have associated raw data
- A description of any restrictions on data availability

Image data used in this analysis may be accessible with approval from an institutional review board, University of Michigan, and the mail-order pharmacy. Contact the corresponding author.

Field-specific reporting

Please select the one below that is the best fit for your research. If you are not sure, read the appropriate sections before making your selection.

☐ Life sciences ☒ Behavioural & social sciences ☐ Ecological, evolutionary & environmental sciences

For a reference copy of the document with all sections, see nature.com/documents/nr-reporting-summary-flat.pdf

Behavioural & social sciences study design

All studies must disclose on these points even when the disclosure is negative.

Study description	This study used a mixed methods approach in evaluating the performance of a ResNet-18 model to predict the shape, color, and national drug code (NDC) labels for images showing a top-down view inside a prescription medication bottle. The data were split into training, validation, and test sets. We report on the results of the test set. An error analysis of misclassified NDC labels by the ResNet-18 model was performed to quantify the physical attributes of pills that were misclassified.
Research sample	Sample of medication images from a single mail-order pharmacy in the United States.
Sampling strategy	1 year worth of images was obtained. No sample size calculation was performed since all images from that year were included and available to the research team. Since the model was pre-trained on Imagenet, the dataset was deemed to be large enough to fine-tune the model with the medication image data.
Data collection	Images were obtained from a mail-order pharmacy. The images were taken during the fill process of a medication dispensing robot. The top-down view inside the prescription vial was shared with the University of Michigan.
Timing	2018 calendar year
Data exclusions	No data were excluded from the analyses.
Non-participation	N/A
Randomization	N/A

Reporting for specific materials, systems and methods

We require information from authors about some types of materials, experimental systems and methods used in many studies. Here, indicate whether each material, system or method listed is relevant to your study. If you are not sure if a list item applies to your research, read the appropriate section before selecting a response.

Materials & experimental systems

n/a	Involved in the study	
☒	☐	Antibodies
☒	☐	Eukaryotic cell lines
☒	☐	Palaeontology and archaeology
☒	☐	Animals and other organisms
☒	☐	Human research participants
☒	☐	Clinical data
☒	☐	Dual use research of concern

Methods

n/a	Involved in the study	
☒	☐	ChIP-seq
☒	☐	Flow cytometry
☒	☐	MRI-based neuroimaging
